# Holocranohistochemistry enables the visualization of α-synuclein expression in the murine olfactory system and discovery of its systemic anti-microbial effects

**DOI:** 10.1007/s00702-017-1726-7

**Published:** 2017-05-05

**Authors:** Julianna J. Tomlinson, Bojan Shutinoski, Li Dong, Fanyi Meng, Dina Elleithy, Nathalie A. Lengacher, Angela P. Nguyen, Greg O. Cron, Qiubo Jiang, Erik D. Roberson, Robert L. Nussbaum, Nour K. Majbour, Omar M. El-Agnaf, Steffany A. Bennett, Diane C. Lagace, John M. Woulfe, Subash Sad, Earl G. Brown, Michael G. Schlossmacher

**Affiliations:** 1 Program in Neuroscience, Ottawa Hospital Research Institute, Ottawa, ON Canada; 2 University of Ottawa Brain and Mind Research Institute, Ottawa, ON Canada; 3 Department of Biochemistry, Microbiology and Immunology, Faculty of Medicine, University of Ottawa, Ottawa, ON Canada; 4 Department of Pathology and Laboratory Medicine, Faculty of Medicine, University of Ottawa, Ottawa, ON Canada; 5 Department of Medical Imaging, The Ottawa Hospital, Ottawa, ON Canada; 6 Department of Cellular and Molecular Medicine, Faculty of Medicine, University of Ottawa, Ottawa, ON Canada; 7 Division of Neurology, Department of Medicine, Faculty of Medicine, The Ottawa Hospital, Ottawa, ON Canada; 8 Department of Neurology, University of Alabama, Birmingham, AL USA; 9 Division of Medical Genetics, Department of Medicine, University of California San Francisco, San Francisco, CA USA; 10 Neurological Disorders Research Center, Qatar Biomedical Research Institute, Hamad Bin Khalifa University, Qatar Foundation, Doha, Qatar; 11 University of Ottawa, 451 Smyth Road, RGH #1464, Ottawa, ON K1H 8M5 Canada; 12 Ottawa Hospital Research Institute, Ottawa, ON Canada; 13 Faculty of Medicine, Department of Radiology, University of Ottawa, Ottawa, ON Canada

**Keywords:** Histology, Parkinson disease, Alzheimer disease, Synucleinopathy, Neuropathology, SNCA/α-synuclein, MAPT/tau, APP/Aβ, Genome, Susceptibility, Exposome, Inoculation, Infection

## Abstract

Braak and Del Tredici have proposed that typical Parkinson disease (PD) has its origins in the olfactory bulb and gastrointestinal tract. However, the role of the olfactory system has insufficiently been explored in the pathogeneses of PD and Alzheimer disease (AD) in laboratory models. Here, we demonstrate applications of a new method to process mouse heads for microscopy by sectioning, mounting, and staining whole skulls (‘holocranohistochemistry’). This technique permits the visualization of the olfactory system from the nasal cavity to mitral cells and dopamine-producing interneurons of glomeruli in the olfactory bulb. We applied this method to two specific goals: first, to visualize PD- and AD-linked gene expression in the olfactory system, where we detected abundant, endogenous α-synuclein and tau expression in the olfactory epithelium. Furthermore, we observed amyloid-β plaques and proteinase-K-resistant α-synuclein species, respectively, in cranial nerve-I of *APP*- and human *SNCA*-over-expressing mice. The second application of the technique was to the modeling of gene–environment interactions in the nasal cavity of mice. We tracked the infection of a neurotropic respiratory-enteric-orphan virus from the nose pad into cranial nerves-I (and -V) and monitored the ensuing brain infection. Given its abundance in the olfactory epithelia, we questioned whether α-synuclein played a role in innate host defenses to modify the outcome of infections. Indeed, *Snca*-null mice were more likely to succumb to viral encephalitis versus their wild-type littermates. Moreover, using a bacterial sepsis model, *Snca*-null mice were less able to control infection after intravenous inoculation with *Salmonella typhimurium*. Together, holocranohistochemistry enabled new discoveries related to α-synuclein expression and its function in mice. Future studies will address: the role of *Mapt* and mutant *SNCA* alleles in infection paradigms; the contribution of xenobiotics in the initiation of idiopathic PD; and the safety to the host when systemically targeting α-synuclein by immunotherapy.

**Electronic supplementary material:**

The online version of this article (doi:10.1007/s00702-017-1726-7) contains supplementary material, which is available to authorized users.

## Introduction

Staging of Lewy pathology in typical, late-onset Parkinson disease (PD) led to the ‘dual hit hypothesis’, proposed by Braak and Del Tredici, in which the disease process begins in the olfactory system and/or the gastrointestinal tract several years before any motor symptoms appear (reviewed in Del Tredici and Braak 2016). Despite some criticism raised regarding the wider applicability of the proposed staging system to all cases of typical PD, their classification scheme seems to properly account for the long, prodromal phase of PD, during which hyposmia and constipation are common features (Berg et al. 2015). In accordance, studying the olfactory system and the role of disease-linked genes expressed in it may provide important insights into disease etiology. However, the olfactory system remains understudied, including in routine laboratory models of PD pathogenesis.

Typical, late-onset (i.e., ‘idiopathic’) PD is thought to be caused by a combination of genetic susceptibility coupled to unknown environmental triggers that may be toxin-based or microbial in nature, as well as by sustained tissue responses, effects of gender, and the passage of time (Kitada et al. 2012; Schlossmacher et al. 2017). These factors interact to bring about the progressive demise of dopamine neurons in the human *Substantia nigra* and other brainstem nuclei. The olfactory and gastrointestinal systems lay at the interface between the host and his/her environment, and could serve as sites of exposure to environmental disease initiating factor(s) (reviewed by Rey et al. 2016). The study of interactions between the exposome and genome at these sites in laboratory models of PD has been lacking.

The olfactory epithelium (OE) rests on the lamina propria in the nasal cavity and is comprised of olfactory receptor neurons (ORNs), support cells, regeneration-competent basal cells, and embedded mucus-producing cells. There, ORNs bridge the environment with the brain for the purpose of smell signalling: their dendrites extend toward the ethmoid sinus and their axons form cranial nerve (CN)-I bundles within the lamina propria. These bundles then traverse the cribriform plate to synapse with mitral cells of the glomeruli within the OB (recently reviewed in Rey et al. 2016). The potential importance of the OE and insights gained from its functions have been under-appreciated in neurodegeneration research, perhaps given that it is invariably lost, along with CN-I fibers, as a result of the routine dissection techniques used in histological studies of rodents.

Here, we describe a new technique that we termed ‘holocranohistochemistry’, by which the olfactory system can be studied within the intact head of a rodent. For this, we processed formalin-fixed, decalcified and paraffin-embedded mouse heads in preparation of thin sections for a range of histological applications. The technique is amenable to studying the intact olfactory system in addition to other structures outside the central nervous system (e.g., respiratory epithelium and CN-V) and intra-cranial structures, all within the proper anatomical context.

While the applications of this technique are not limited to the study of the olfactory system, our goal in developing this method was twofold. The first was to enable the assessment of expression of PD-linked proteins, such as α-synuclein and tau, within elements of the OE in mice. Although these proteins have been identified in autopsy material of human OE (Arnold et al. 2010; Duda et al. 1999), their presence and possible function(s) in the intact olfactory system of mice have not yet been studied. The second goal in developing this technique was for the purposes of modeling and visualizing PD-linked gene interactions with the environment in the olfactory system in mice and monitoring the ensuing effects on brain health (Kitada et al. 2012; Schlossmacher et al. 2017). As presented herein, the technique of holocranohistochemistry enabled us to visualize and track an infection from the nasal cavity as it spreads to the brain, to monitor local immune responses, and to observe the ensuing organism-wide effects of a viral pathogen. We also explored a possible *Snca* gene–environment interaction using this infectious model and identified a role for endogenous α-synuclein in the host’s innate immune defense, which we validated using a second, bacterial infection paradigm.

## Materials and methods

### Ethics statement

All mouse studies and tissue collections were performed in accordance with protocols approved by the University of Ottawa Animal Care and Veterinary Services Committee. Post mortem, human tissue samples were collected at autopsy and used in accordance with institutional guidelines and following the approval by ethics review boards at participating hospitals.

### Mouse models

Double (dbl)-PAC-transgenic (tg)(*SNCA* ^A53T^)^+/+^; *Snca* ^−/−^ mice, on an FBV/Nx129S6 background have 4 insertions of the human *SNCA* locus, as described (Kuo et al. 2010). For *SNCA* gene-dosage studies, the dbl-PAC-tg(*SNCA* ^A53T^)^+/+^; *Snca* ^−/−^ mice were crossed with *Snca* ^−/−^ on the same genetic background (Kuo et al. 2010) to generate offspring with 2 PAC insertion sites. *Snca* ^−/−^ mice, on a FBV/Nx129S6 background (Kuo et al. 2010), were used for microscopy. For microbiological studies, *Snca* ^−/−^ C57Bl/6J mice [derived from *Snca* ^−/−^ mice described in Cabin et al. (2002) and kindly provided by Dr. M. Farrer] were used. *Mapt* ^−*/*−^ mice, on a C57Bl/6x129SvJ background, were previously described (Dawson et al. 2001). Human *APP*-over-expressing, tg mice (N5 TgCRND8), on a C57Bl/6xC3H/HeJ background, have also been described (Granger et al. 2016; Chishti et al. 2001).

### Magnetic resonance imaging

**Fig. 1:**
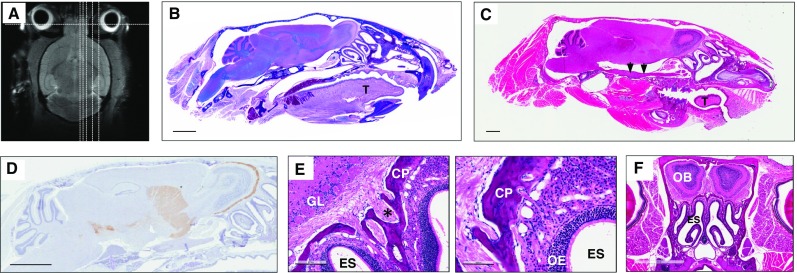
Whole head mounting allows for visualization of the intact olfactory system by routine microscopy. **a** Magnetic resonance image of an adult mouse head with sagittal and coronal lines (*dotted*) depicting examples of levels for sectioning. **b** Luxol fast blue-stained, and **c** haemotoxylin and eosin (H&E)-stained, sagittal head sections (all, 5 μm) from an adult mouse that visualize, among other structures: the brain with an intact olfactory bulb (**b** more medial; **c** lateral), turbine within the ethmoid sinus, the palate, tongue (“T”), alveolar bone (and teeth), an intact olfactory epithelium including cranial nerve (CN)-I fibers as well as CN-V at the base of the skull (**c** *arrows*). **d** Tyrosine hydroxylase immunohistochemistry-based staining of an intact skull section from an adult mouse (at an intermediate level) highlighting the *Substantia nigra* of the midbrain, neostriatum, and dopamine-producing neurons of olfactory glomeruli (*brown staining*). **e** Higher magnification of H&E stained, sagittal section of the olfactory system in an adult mouse. *ES *ethmoidal sinus, *CP* cribriform plate, *GL* glomeruli; *asterisk* denotes axonal bundles of CN-I. **f** H&E staining of an adult mouse skull prepared for coronal sectioning at the level of the olfactory bulb (OB) and turbine/ES, as depicted in **a**. *Scale bars* represent 2 mm (**b**–**d**, **f**); 200 μm (**e**, *left image*); 100 μm (**e**, *right image*)

Mouse brain magnetic resonance imaging (MRI) was performed at the University of Ottawa Pre-Clinical Imaging Core Facility using a 7 Tesla GE/Agilent MR 901. A 2-D, fast-spin-echo sequence (FSE) pulse sequence protocol was employed, with the following parameters: 18 prescribed slices; slice thickness = 0.7 mm; spacing = 0 mm; field of view = 2 cm; matrix = 256 × 256; echo time = 25 ms; repetition time = 4000 ms; echo train length = 8; bandwidth = 16 kHz; 4 averages; and positive fat saturation. The mouse (5 months old) shown in Fig. 1 was euthanized immediately prior to image acquisitions.

### Whole head preparation of mice

When studying adult mice, animals were perfused with phosphate buffered saline (PBS, 10 ml) followed by buffered 10% formalin (10 ml; Fisher Scientific, Ottawa, ON, Canada) via cardiac puncture. Heads were collected by decapitation, the scalp removed, and then fixed by submersion in 10% formalin for 48 h at 4 °C and transferred to 70% ethanol for short-term storage (1–10 days). Bone was decalcified by submersion in 12.5% formic acid for 5 days and heads rinsed with running tap water for 2–3 h. Tissue specimens were dehydrated by sequential submersions in 60, 70, 80, 90% (1 × 1 h, respectively), and 100% ethanol (4 × 1 h), followed by 2 × 1 h in xylene/toluene and 4 h in paraffin solutions prior to embedding. For murine pups (P1–P12), animals were decapitated, the blood drained on an absorbent pad, and heads fixed in 10% formalin for 24 h. Subsequent processing was done in the same manner as above, with the exception that decalcification was reduced to 3 days due to lesser ossification of skull bones.

Paraffin-embedded blocks were sectioned at 5 μm each and mounted onto glass slides. To visualize the olfactory system, we optimized sectioning at three levels, with level 1 being medial, approximately 1.0 mm from the midline. Each lateral block (levels 1–6) was cut at a minimum of 50 μm distance. Mounted head sections were dried at 37 °C for 48 h.

Ossified bone tissue, although decalcified, can interfere with adherence of distinct regions of the skull to the slide. To minimize artefacts, new batches of slides were vetted to ensure proper adherence; increased caution was taken in the processing of sections during automated, histological development and manual (as well as automated), immunohistological processing, and in particular, when proteinase-K digestion was performed. There, slides were re-baked at 37 °C for 10 min prior to processing for staining.

### Immunohistochemistry and indirect immunofluoresence

Immunohistochemistry- and indirect immunofluorescence-based microscopy was performed as previously described (Schlossmacher et al. 2002; Schlossmacher and Shimura 2005) using routine protocols. For proteinase-K digestion, slides were incubated with 0.2 mg/ml proteinase-K (Sigma-Aldrich, Oakville, ON, Canada) for 3 min at room temperature in 20 mM Tris–HCl, pH 8.0 prior to antigen retrieval. Immunohistochemical slides were developed with the Vectastain Elite kit (Vector Laboratories Inc, Burlingame, CA, USA), counterstained with hematoxyline and scanned using an Aperio ScanScope Console (Leica Biosystems, Concord, ON, Canada). Aperio ImageScope software was used to capture the regions of interest from the entire head mounted section. Fluorescent images were captured using a Zeiss LSM 510 META/AxioVert 200 Confocal Microscope.

For development, the following primary antibodies were used: anti-tyrosine hydroxylase (anti-TH by Millipore, Etobicoke, ON, Canada; 1:5000); hSA4 (a non-commercial, polyclonal Ab, that was raised and affinity-purified against recombinant, full length, human α-synuclein as described (Mollenhauer et al. 2008); 1:250–1:1000; [of note, its monoclonal Ab derivative is commercially available as MJFR1 from Abcam and its staining characteristics were described in Gray et al. (2014)]; LB509 (18-0212; Zymed, ThermoFisher, Ottawa, ON, Canada; 1:100–1000); Syn-1 (BD Biosciences, Mississauga, ON, Canada; 1:100–1000); OT21C (a kind gift from Dr. Jaap Middledorp; 1:5000); anti-OMP (5411-10001, Wako Chemicals, Richmond, VA, USA; 1:300–400); Tau-1 (Millipore, Etobicoke, ON, Canada; 1:300); Amyloid-β, clone 4G8 (SIG-39220, Covance, Princeton, NJ, USA; 1:400); polyclonal, affinity-purified anti-reovirus-T3D [non-commercial; described in: Gauvin et al. (2013); 1:5000]; and Iba1 (019-19741, Wako Chemicals, Richmond, VA, USA; 1:1000).

### Quantification of α-synuclein

Mice were perfused with PBS (10 ml) by cardiac puncture, and isolated brains were further dissected to collect the olfactory bulbs and forebrain (for quantification in pups of ages P1–P42, the whole brain was used). Brain tissues were homogenized in TXS buffer [50 mM Tris–HCl, pH 7.4, 140 mM NaCl, 0.5% Triton X-100, 1× proteinase inhibitor cocktail (Roche Diagnostics, Indianapolis, IN, USA)]. Lysates were equalized for total protein concentration and α-synuclein was quantified using sandwich ELISAs for total- and soluble oligomeric α-synuclein, as previously described (Mollenhauer et al. 2008; Cullen et al. 2011; Vaikath et al. 2015).

### Olfactory behavioural testing

Olfactory testing was performed at the University of Ottawa Behavioural Core Facility. The Odour Detection test was carried out as described (Petit et al. 2013). For this test, anise and vanilla extracts (Club House, London, ON, Canada) were diluted in distilled water as the olfactory cue and each mouse was tested at each concentration in 2 min sessions with 1 min inter-trial-interval (ITI). The habituation–dishabituation test was performed as described (Yang and Crawley 2009). For this test, the non-social cues were pure almond and imitation banana extracts (Club House, London, ON, Canada) diluted 1:100 in distilled water; the social cues were swabs taken from unchanged (3 days) cages that housed mice of the opposite sex. Mice were tested in 2 min sessions with 1 min ITI.

### Infection paradigms

#### Reovirus serotype-3 Dearing strain (T3D) studies

Live T3D virus was prepared in L929 cells as described (Gauvin et al. 2013). For inoculation, mouse pups (i.e., 20–30 h of age; littermates), were anesthetized using 3% isoflurane in O_2_, and 1.7 × 10^5^ plaque-forming units (PFU) of viral preparation diluted in PBS (10 μl total) were placed on the nose pad for aspiration into the respiratory tract and nasopharynx. For mock-treated controls, 10 μl of conditioned L929 media similarly diluted in PBS was used. Pups were then returned to their mother, as described previously (Gauvin et al. 2013). For survival assay, a moribund state of ensuing encephalitis was selected as the humane endpoint (as approved by the animal care ethics review board), upon which mice were euthanized (by CO_2_ narcosis) and ear tissue was collected for genotyping. For determination of viral load in their brains, separate cohorts of mice were collected at 10 day post-inoculation (dpi); their whole brains were isolated and homogenized in nine volumes (weight/volume) of PBS. The plaque-forming assay was performed by infecting L929 cells with serially diluted brain lysates and overlaid with agar media, as described previously (Gauvin et al. 2013).

#### *Salmonella typhimurium* studies

Eight week-old wild-type (WT) and *Snca* ^−*/*−^ littermates were inoculated with 200 colony-forming units (CFU) of *Salmonella enterica* subsp*. enterica* serovar Typhimurium SL1344 (abbreviated as *S. typhimurium*) by intravenous injection into the tail vein, as previously described (Shutinoski et al. 2016). Mice were sacrificed at 5 dpi, and spleens were collected. Splenic bacterial load was quantified by plating serial dilution of lysates onto agar plates.

## Results

### Visualization of intra- and extra-cranial structures by microscopy of murine heads

Holocranohistochemistry is a new whole skull mounting technique that enables histological studies of the intact olfactory system, brain, and associated nerves, and of contiguous systems in mice by routine microscopy. For this, intact, formalin-fixed mouse heads were collected, the scalp was removed from the snout to the occiput, and the skull bones were decalcified by submersion in 12.5% formic acid. The heads were then paraffin-embedded following routine procedures and individually sectioned (5 μm) in either a sagittal or coronal plane, as informed by earlier MRI studies of anatomical landmarks (Fig. 1a). Given our interest in studying an intact olfactory system, we optimized the generation of multiple, serial levels beginning medially, sectioning approximately 1 mm parallel to the midline, as identified by the sagittal suture (an example is shown for level 1 in Fig. 1b) and continuing through the olfactory bulb (OB) in up to 5–7 parallel ‘blocks’ that are at least 50 μm apart (example for level 3 shown in Fig. 1c). Luxol fast blue (LFB; Fig. 1b) as well as haemotoxylin and eosin (H&E; Fig. 1c) staining of these sections revealed among others, the preserved turbine structures of the nasal cavity, including both respiratory and olfactory epithelia on their surface, both of which are exposed to air flow within the ethmoid sinus (ES; Fig. 1b, c).

Using whole skull sectioning, the olfactory bulb itself is structurally preserved, allowing the interrogation of the glomeruli that are otherwise anatomically disrupted or lost during routine brain dissections and isolation. An example of the value of preserving these structures is the visualization of the intact dopaminergic system in a whole skull section (cut at the intermediate level 2; Fig. 1d), in which immunostaining for tyrosine hydroxylase (TH) revealed the *Substantia nigra* in the inferior midbrain, the neostriatum, and the abundance of dopamine-producing neurons in the glomeruli of the OB. TH protein has been shown to be highly expressed in interneurons that modulate synaptic activity at the interface between axons of olfactory receptor neurons and mitral cells, where, for example, dopamine has recently been shown to be involved in D2 receptor-mediated suppression of neurotransmission (Hsia et al. 1999; Banerjee et al. 2015).

Higher magnification of the olfactory system within the nasal cavity (Fig. 1e) shows the surrounding ethmoid sinus and the olfactory epithelium itself; the latter comprises olfactory receptor neurons (ORNs), non-neural support cells, and mucus-producing cells [for recent review and structural details, see Rey et al. (2016)] as well as axons of ORNs that form CN-I. CN-I fibers traverse the lamina propria to form bundles and cross the cribriform plate to enter the cranium, where they synapse with relay neurons of glomeruli in the OB. These structures can also be visualized by serial, coronal sections of the same region (Fig. 1f, the plane of which is depicted as the horizontal line in Fig. 1a).

In accordance with the level of sagittal (or coronal) sectioning, all other extra-cranial as well as intra-cranial components of the adult, murine head can be visualized in their anatomically correct positions, including (but not limited to) maxillary and mandibular bone elements, such as teeth (Fig. 1b, c), the nasal sinus system and its conchae, the base of the skull, the oral cavity including the tongue and palate, lips including subcutaneous and epidermal appendages, neurites of CN-V, the visual system including eyes, eye muscles (and CN-II, -III, -IV, and -VI), salivary, thyroid and pituitary glands, arterial, venous and lymphatic structures, the dura mater and arachnoid (and related spaces created by them), the brain in its entirety, as well as upper portions of the cervical spinal cord (Fig. 1b, c). Most of these structures are lost or compromised when whole brains are dissected from the skull or are studied in isolation from the intact brain.

### α-Synuclein is abundantly expressed in olfactory receptor neurons including their dendrites

**Fig. 2:**
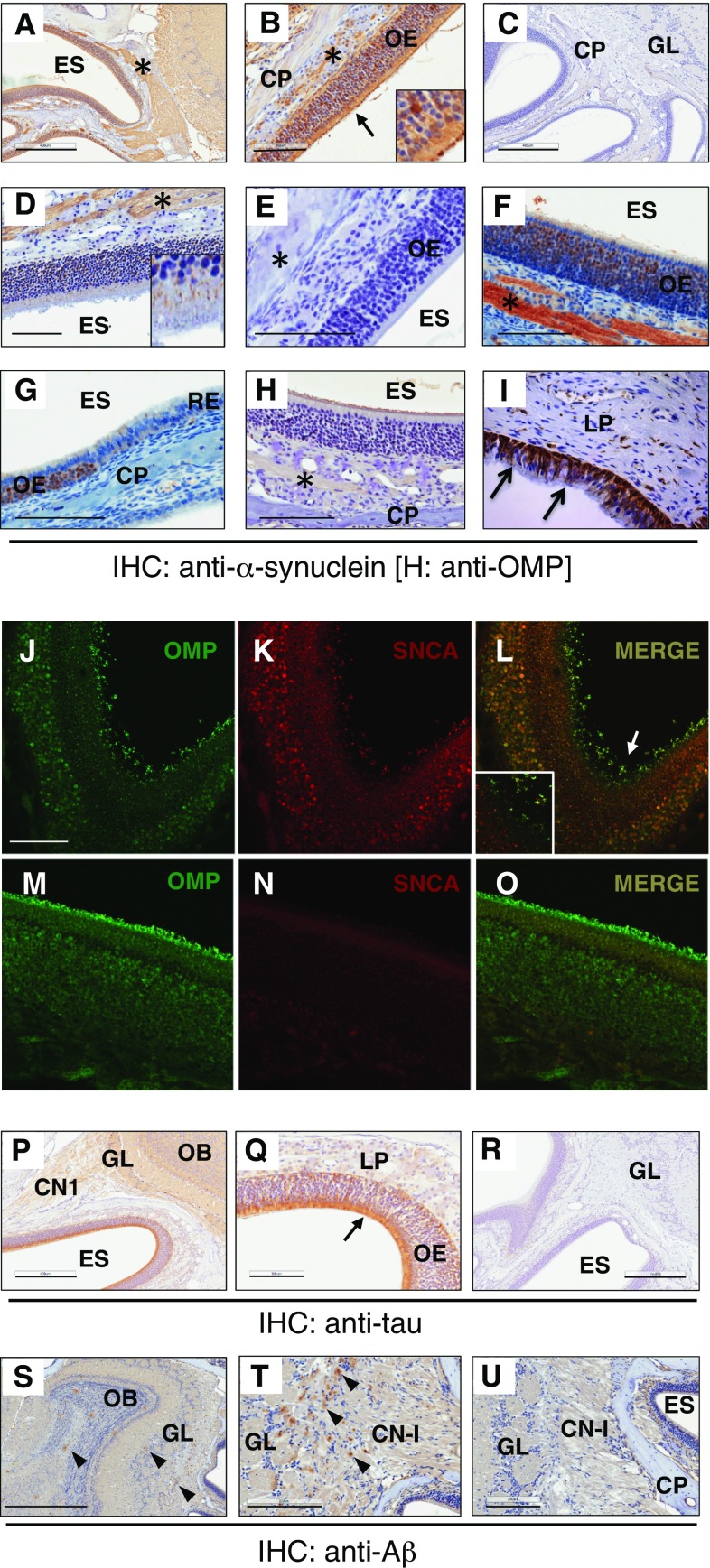
α-Synuclein and tau are abundant constituents of the olfactory epithelium. **a**–**g** Immunohistochemistry (IHC)-based staining of α-synuclein in sections of whole skull preparations from adult mice reveals its specific expression in olfactory receptor neurons and CN-I (*asterisk*). Detection of human α-synuclein expression in adult dbl-PAC-tg(*SNCA* ^A53T^)^+/+^; *Snca* ^−/−^ (**a**, **b**, **d**), absent in *Snca*-null (**c, d**) mice using antibodies (Ab) hSA4 (**a**–**c**) and LB509 (**d**, **e**). *Insets* in **b** and **d** are of olfactory epithelium (OE) at higher magnification and reveal α-synuclein expression in the dendrites of olfactory receptor neurons. **f, g** Endogenous, murine α-synuclein expression in the OE and CN-I, but not the respiratory epithelium (RE) of adult wt mice (Ab, Syn-1). **h** Typical staining of olfactory marker protein (OMP) in the OE of an adult wt mouse. **i** Expression pattern of α-synuclein in paraffin-embedded sections of human OE tissue collected at autopsy (Ab, LB509). Indirect immunofluorescence-based microscopy to co-label α-synuclein and OMP in olfactory receptor neurons in adult dbl-PAC- tg(*SNCA* ^A53T^)^+/+^; *Snca* ^−/−^ (**j**–**l**) versus *Snca*-null (**m**–**o**) mice. *Arrows* in **l** and *inset* denote co-labelled dendritic knobs of olfactory neurons projecting into the ethmoid sinus. No double labelling is seen in **o**, as expected. IHC staining for specific, endogenous tau expression in the olfactory epithelium (OE) of adult wt (**p**, **q**) and *Mapt*-null (**r**) mice. IHC-based staining for amyloid-beta protein (Aβ) in sagittal sections of 6-month-old APP-transgenic (**s**–**t**) and wt (**u**) mice reveals Aβ-positive plaques in CN-I and throughout the olfactory bulb (*arrowheads*) in the former. *ES* ethmoid sinus; *CP* cribriform plate, *GL* glomeruli, *asterisk* denotes CN-I, *LP* lamina propria. *Scale bars* represent 1 mm (**s**); 400 μm (**c**, **p**, **r**); 200 μm (**a**); 100 μm (**b**, **d**–**j**, **q**, **t**, **u**)

Our first goal in the development of this technique was to enable the visualization of PD-linked gene expression in the olfactory system of mice. Specifically, given the role of α-synuclein in PD pathogenesis and the early detection of Lewy pathology in the OB during the prodromal phase of disease as per Braak and Del Tredici’s classification scheme (and the associated hyposmia), we probed for α-synuclein expression in the olfactory system in newborn (as young as P3) and aged (up to 24-month-old) mice. As expected, we observed staining of normal α-synuclein throughout the entire brain across the lifespan of the mouse (not shown). Of particular interest to us was the abundant expression of α-synuclein that was detected by multiple antibodies within ORNs in mice (Fig. 2a–g), which was also visualized in human OE tissue collected at autopsy (Fig. 2i), as previously reported (Duda et al. 1999; Arnold et al. 2010). Intriguingly, we found that α-synuclein, which is largely known as a ‘pre-synaptic protein’, was also present in the dendrites of olfactory neurons at the interface of the ORE and the airflow of the ethmoid sinus (see insets of Fig. 2, panels b, d, l). As expected, α-synuclein was found abundantly in the neuronal cell bodies and axons that form CN-I. The protein is also highly expressed in the glomeruli and throughout the OB. This observation was true for both human and murine α-synuclein expressed in respective mouse models [dbl-PAC-tg(*SNCA* ^A53T^)^+/+^; *Snca* ^−/−^ (Kuo et al. 2010) (Fig. 2a–e) and wild-type (wt) mice] (Fig. 2f, g). *SNCA/Snca* gene expression was absent in non-neural, respiratory epithelial cells (Fig. 2g). Similar anatomic features were found when multiple anti-α-synuclein antibodies (Abs) were used for immunostaining, including polyclonal hSA4 (panels a–c) and monoclonal Abs LB509 (panel d), Syn-1 (panels e, g) and OT21C (Supplemental Fig. 1). *Snca* ^−/−^ sections were used to show signal specificity. Expression of α-synuclein in the dendrites and throughout the cytoplasm of ORNs within the olfactory epithelium as well as in axons of CN-I and glomeruli of the OB was confirmed by co-labelling with olfactory marker protein (OMP; Fig. 2h; for co-labelling, see Fig. 2j–o).

α-Synuclein is a highly abundant, neuronal, and red blood cell (Scherzer et al. 2008) protein. Higher order, pre-fibrillar isoforms are considered to be neurotoxic (Walsh and Selkoe 2016) and are associated with PD pathogenesis. A pool of modified species can be revealed histologically by digestion with proteinase-K. Importantly, the new holocranohistochemistry technique was compatible with proteinase-K treatment, although the tissues were more fragile compared to routinely processed isolated whole brain sections. Using holocranohistochemical analysis of aged, adult mice (8- and 24-month), we found specific, proteinase-K-resistant α-synuclein reactivity in CN-I axons and the glomeruli of the olfactory system (which, surprisingly, could also be seen in wt mice). We also confirmed the ability to specifically detect phosphorylated α-synuclein (at Ser129; Wako Chemicals Ab) in the brain using this technique; however, we did not observe any specific signal in the OE or CN-I (not shown). Furthermore, under these conditions, we detected no Thioflavin-S (or -T) positivity in any of the neural structures of α-synuclein over-expressing animals (not shown). The cytoplasm and dendritic structures of ORNs did not reveal any proteinase-K-resistant signals by routine microscopy, as could be expected for these abundant, soluble pools of α-synuclein (Supplemental Fig. 1A–G).

### Exploring the effects of *SNCA* allele dosage in the olfactory system

Analysis of protein expression within the olfactory system using whole head mounting can be performed in parallel to other biochemical and/or functional readouts. For example, to determine whether human α-synuclein expression levels and soluble oligomer formation within the olfactory system correlated with altered olfaction in the PAC-tg(*SNCA* ^A53T^); *Snca* ^−/−^ mice, we compared age-matched mice that carried 2 versus 4 insertions of the PAC-transgene encoding the human *SNCA* locus (in the absence of murine *Snca*) (Kuo et al. 2010). While the whole skull mounting technique readily revealed proteinase-K-resistant, α-synuclein-positive reactivity in CN-I and OB glomeruli, the sensitivity of the technique was insufficient to reveal reliable detection of *SNCA* copy number-dependency in either the 8- or 24-month-old mice (representative images of 8-month-old mice are shown in Supplemental Fig. 1). In contrast, ELISA-based quantification for total and soluble oligomeric α-synuclein levels in 16 month-old animals reliably showed the expected *SNCA* gene-dose-dependency in homogenates of the OB and forebrain (Suppl. Fig. 1j, k). Olfactory dysfunction has been described in the prodromal state of PD (Berg et al.), and reported in tg-*SNCA* mouse models (Fleming et al. 2008; Petit et al. 2013). Intriguingly, in our mice, increased α-synuclein expression in the olfactory system correlated with a significant, *SNCA* gene-dose-dependent difference in olfactory function, determined using the olfactory habituation-dishabituation test (Yang and Crawley 2009). Specifically, higher *SNCA* dosage correlated with decreased measure of olfaction that was significant in the context of a social cue; however, these odour detection tests confirmed that primary olfaction, odour discrimination, and memory of olfactory cues were unaffected by higher *SNCA* load (Supplementary Fig. 1l, m).

With specific relevance to possible applications to α-synuclein biomarker-related work in neurodegenerative disorders (reviewed in Mollenhauer et al. 2016b), we also observed some *SNCA* gene-dose-dependent differences in physiological α-synuclein expression in pre-synaptic nerve endings of hair follicles, at neuromuscular junctions of striated muscle fibers in the tongue, and neurites in the salivary glands (not shown). We concluded from these collective results that our current protocol of whole skull mounting permitted an anatomic structure-based (qualitative) expression analysis; a more quantitative assessment of α-synuclein metabolism in the olfactory system and other neural structures of the skull (for diagnostic purposes) requires further analyses.

### Holocranohistochemistry permits visualization of normal tau expression and amyloid-β protein plaque formation in the olfactory system

To further study the applicability of whole head mounting to other models of neurodegeneration and given the abundance of physiological α-synuclein in ORNs of mice and humans, we explored a second gene, which is linked to both PD and AD. *MAPT* encodes 6 isoforms of tau, a microtubule-associated protein largely seen in the cytoplasm and axons (Wang and Mandelkow 2016); in addition to being a key player in AD pathogenesis, variants at the *MAPT* locus represent the second most commonly associated risk factor for PD by GWAS analysis (after changes at the *SNCA* locus) (Edwards et al. 2010). Pathological tau species have previously been found by microscopy in the cytoplasm of ORNs from both AD and neurologically intact subjects (Arnold et al. 2010). We, therefore, investigated endogenous, total tau expression in adult mice, and found it to be highly abundant throughout the olfactory epithelium (Fig. 2p, q). This observation was specific, as *Mapt*-null mice showed no immunoreactivity (Fig. 2r). Its localization included the cytoplasm of ORNs that contained α-synuclein (see above) as well as the axonal bundles of CN-I, as expected. The role of physiological tau in the olfactory epithelium, e.g., in odour processing, and how its metabolism may relate to disease pathogenesis (including of PD and AD) remain unknown and warrant further study.

Like tau, Aβ protein is an essential factor in AD pathogenesis (Selkoe 2007), and has been reported to be expressed in resected human olfactory epithelium, where its presence correlated with the severity of AD (Arnold et al. 2010). Using the N5 TgCND8 mouse model, which expresses a double-mutant isoform of the human amyloid precursor protein (APP) and generates amyloid plaques in the brain by 3 months of age (Chishti et al. 2001; Granger et al. 2016), we probed for Aβ expression and the presence of neuritic plaque formation in whole skull mounts. In the brain, we readily visualized the presence of Aβ plaques using H&E and Thioflavin-T staining (not shown), which was confirmed by immunohistochemistry using antibody 4G8 to human Aβ (Fig. 2s–u). While the presence of Aβ plaques throughout the brain including in the OB (Fig. 2s, t) was expected (Chishti et al. 2001), the whole skull mounting technique enabled us to also detect an even higher density of plaque formation within the axonal bundles of CN-I. Together with the findings of α-synuclein and tau expression described above, holocranohistochemistry allows us to address the normal and potentially pathological roles of these three pivotal proteins in commonly used mouse models of neurodegenerative diseases. The technique permits the visualization of their expression throughout an anatomically intact olfactory system from the nasal cavity (including the vomeronasal organ at its base; not shown) to higher association cortices.

### Modeling a natural course of infection that begins inside the nasal cavity

**Fig. 3:**
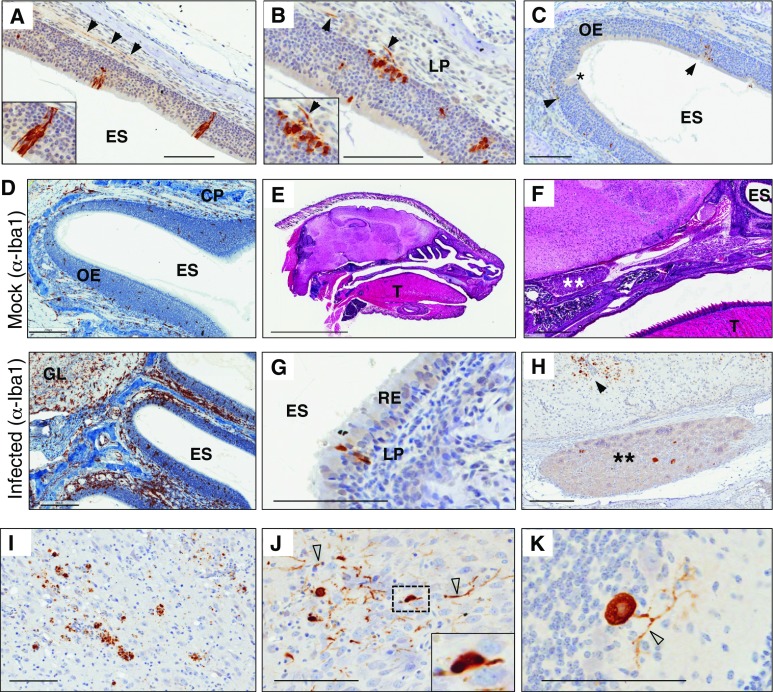
Tracking a viral infection by holocranohistochemistry following nasal inoculation. **a**–**c** Representative IHC-based, microscopic images of reovirus [serotype-3 Dearing (T3D)] infection of olfactory receptor neurons and axons of cranial nerve (CN)-I following nasal inoculation of 1–2-day-old wt mice using an anti-reovirus-T3D antibody (*brown*). Higher magnification of the olfactory epithelium (OE) in insets (**a, b**) reveals reovirus protein expression in dendrites and throughout ORNs as well as in basal cells sitting above the lamina propria; images are representative of viral infection observed 1–5 dpi. **c** Lower magnification of the OE highlighting residual viral protein expression (*arrowheads*) and the repair of previously infected neurons (*asterisk*), as routinely observed 5–10 dpi (*arrowheads*). **d** Representative IHC-based images of the olfactory system in mock-treated and reovirus-T3D infected, wt mice to monitor microglia activation and macrophage recruitment (anti-Iba1) 3 dpi. Note the robust immune response in areas of the OE, CN-I and adjacent olfactory bulb. H&E staining of sagittal sections of a 3-day-old, wt mouse processed for whole skull mounting (**e**). **f** Higher magnification of 3-day-old mouse pup highlighting the relay ganglion (“*Gasseri*”) of CN-V (*asterisk*); *T* tongue, *ES* ethmoid sinus. **g** Reovirus-T3D infection of a respiratory epithelial (RE) cell (*brown*). **h** Reovirus infection of neurons outside the brain within the ganglion* Gasseri* of CN-V (*asterisk*) and neurons within the brain (*arrowhead*), as shown in a wt mouse 11 dpi. Representative IHC-based images of reovirus-T3D infected neurons in the central nervous system of a wt mouse, where viral titres peak 8–9 dpi, thereby leading to lethal encephalitis. **i** Examples for viral infection of neurons in the thalamus, **j** the midbrain, and **k** of a Purkinje cell of the cerebellum are shown by anti-reovirus-T3D staining. *Inset* in **j** shows reovirus-T3D Ab-positive, cytoplasmic inclusions in an infected neuron. *Open arrow heads* denote dystrophic neurites. *Scale bars* represent 5 mm (**e**); 500 mm (**f**); 200 μm (**d**, **h**); 100 μm (**a**–**c**, **g**, **i**–**k**)

The second goal of developing this technique applied to the study of gene–environment interactions that begin in the olfactory system in PD-related mouse models. Specifically, we focused on environmental hits that are microbial in nature, and as a first step in building these models, we established an ‘infectious challenge’ paradigm in wt mice. To this end, we inoculated newborn mice with a virulent, ubiquitously present microbe using a nasal delivery paradigm that leads to systemic infection with subsequent death from encephalitis (Gauvin et al. 2013). Our ongoing studies for the role of PD-associated genes in the immune system [foremost *LRRK2* (Hakimi et al. 2011)] prompted us to establish this experimental paradigm to model a natural route of infection. Reovirus-T3D is a neurotropic respiratory-enteric-orphan virus that causes lethal brain infection in suckling mice, commencing around 9 days post-inoculation (dpi) (described in Gauvin et al. 2013). Specifically, the virus is administered onto the nose pad of 1–2-day-old pups, from which it is inhaled and swallowed into the respiratory and intestinal tracts, respectively. The viral titre peaks in the lung 3 dpi, and from there (as well as the intestinal tract) spreads systemically via the bloodstream to infect peripheral organs and tissues (Gauvin et al. 2013). Replication in most tissues is maximal at early time points (3–5 dpi) and decreases thereafter; however, viral titres in the brain peak at 8–9 dpi, thereby leading to encephalitis and death in the majority of animals. While most of the virus is distributed to other organs via a hematogenous spread, it also infects ORNs and, on rare occasions, respiratory epithelial cells in the nasal cavity (Fig. 3). We, therefore, chose the virulent reovirus-T3D inoculation model (with its olfactory and gastrointestinal involvement) as a platform to restage a ‘two-site entry’ paradigm (i.e., via the nose and gut) for an environmental pathogen.

Immunohistochemical staining of sections prepared by whole skull mounting with antibodies to purified T3D reovirus permitted the visualization and tracking of this microbial infection in ORNs. There, viral proteins could be seen moving from the olfactory epithelium into axons of CN-I (Fig. 3a–c). Prominent infection of the ORNs was consistently seen at 1–5 dpi, after which the repair of damaged epithelium was monitored (Fig. 3c); the replacement of lysed ORNs and repair of epithelial integrity in these young mice appeared to be complete by 7–10 dpi.

In parallel, a strong immune response was elicited after infection of the olfactory epithelium to clear the virally infected ORNs and their affected axons (including when these had traversed the cribriform plate). In rare cases, we detected viral protein expression in other regions of the olfactory bulb (in >50 infected skulls analyzed by us by ≥11 dpi). Indeed, by 3 dpi, we observed a robust signal for Iba1-positive, activated microglia within the OB and in infiltrating macrophages that had been recruited to all layers of the OE, the lamina propria, and CN-I (Fig. 3d; compare both panels). Given that the entire skull is visualized, other regions of the nervous system can be explored concurrently: for example, infected relay neurons within the ganglion (*Gasseri*) of CN-V, which is positioned at the base of the skull, can be seen by 3–5 dpi (Fig. 3f, h). Viral expression in these neurons resulted from the rare infection of cells within the respiratory epithelium of the nasal cavity (Fig. 3g) and the subsequent propagation of virions within dendritic processes of the trigeminal nerve (CN-V). The spread of infection to the brain via CN-I (and -V) as well as through the bloodstream underlies the development of encephalitis in this model. Of note, infection of the brain by reovirus-T3D from peripheral, intramuscular injection has also been shown to involve a haematogenous spread (reviewed in Schiff et al. 2007; Boehme et al. 2013). The relative contribution of nasal epithelia- versus blood-derived reovirus in the seeding of the CNS in our paradigm has not yet been fully determined.

Murine pups succumb to reovirus-T3D induced encephalitis, where viral titres in the brain peak at 8–9 dpi (Gauvin et al. 2013). Death is infrequently seen before day 8 but will occur as late as 25 dpi (see below). As highlighted in serial sections of whole head mounts from pups at 10 dpi, once present in the brain parenchyma, reovirus-T3D infects multiple, vulnerable nuclei, thereby leading to prominent neuronal infection in the superior and inferior colliculi (tectum) of the midbrain (Fig. 3i), nuclei of the thalamus (Fig. 3j), regions of the pons and medulla oblongata, the hippocampus (not shown), and the cerebellum (Fig. 3k). In Fig. 3, panels i, j show representative images that highlight the pathological spectrum of infected neurons, from dystrophic neurites (arrows), to cellular debris due to neuronal death, and intriguingly, to the presence of reovirus protein-positive, cytoplasmic inclusions in some infected cells (inset Fig. 3j). Of note, under these conditions, the virus infrequently infects glia directly, but can be seen in phagocytic microglia following neuronal death (not shown).

### Holocranohistochemistry informs the modeling of complex disease in mice to test PD gene functions: example of an interaction between genetic susceptibility and an environmental trigger

Holocranohistochemistry has been employed by us to routinely track reovirus-T3D infections and the ensuing immune responses in mouse models carrying modifications in PD-linked genes. Our ultimate goal is to examine interactions between genetic susceptibility (to PD) and environmental triggers as they relate to brain health and possible disease pathogenesis. The physiological role of α-synuclein in the olfactory system beyond odour processing (Suppl. Fig. 1), if any, is unknown. Given the abundance of this protein in ORNs [as well as of β- and γ-synuclein expression (Duda et al. 1999)] (Fig. 2a–o), we asked whether α-synuclein was involved in host responses to and outcome measures of microbial infections. This idea was informed by a recent report that Aβ confers robust, anti-microbial functions in vivo, as does recombinant α-synuclein protein in vitro (Park et al. 2016; Soscia et al. 2010).

**Fig. 4:**
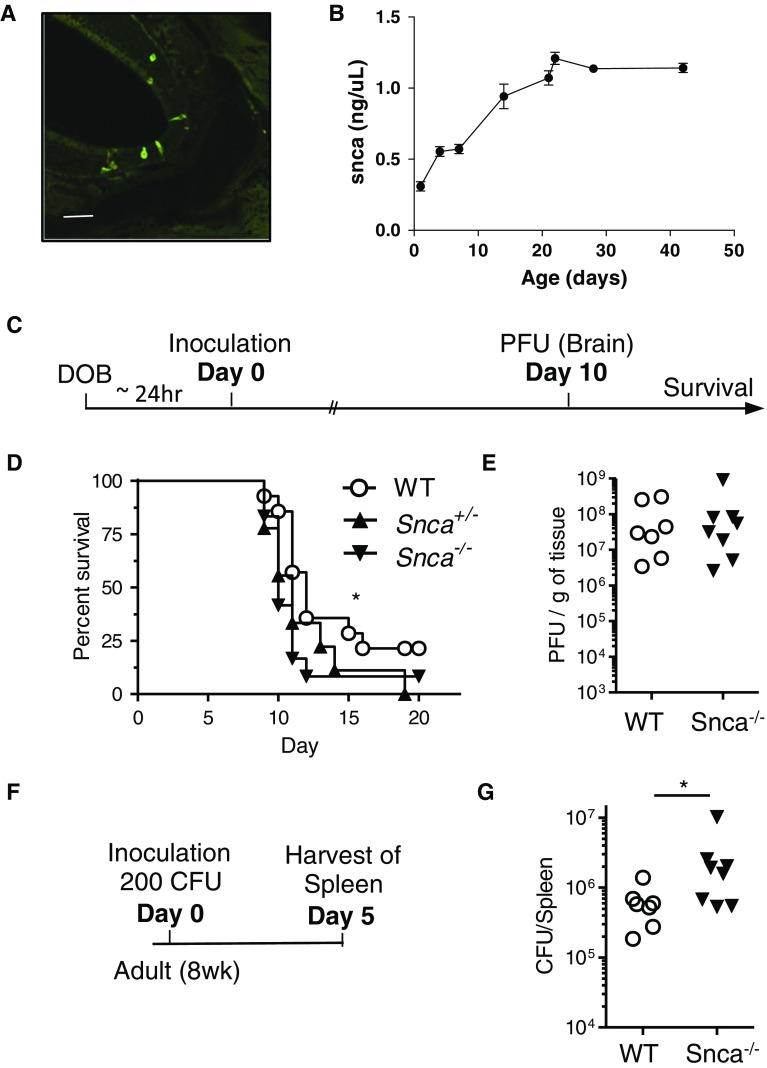
Endogenous α-Synuclein protects the murine host against microbial infections. **a** Representative image of indirect, immunofluorescence-based microscopy of reovirus-T3D infected olfactory receptor neurons 3 dpi. *Scale bar* represents 100 μm. **b** Quantification of endogenous α-synuclein protein concentration in P1 to P45 wt mice. Each time point represents the average of *n* ≥ 3 pups ± SEM. **c** Schema of experimental design to test survival and viral load after reovirus-T3D inoculation. Wild-type (wt) and *Snca*-null (Snca^−/−^) littermates were inoculated via the nose pad with a viral dose of 1.7 × 10^5^ plaque-forming units (PFU). **d** Graph for survival assay. Littermates from heterozygous breeding pairs were used where wt *n* = 14, *Snca* ^+/−^ *n* = 9 (heterozygous); *Snca* ^−/−^ *n* = 12 (male and female). Data were analyzed using the log-rank (Mantel–Cox) test, where *p* = 0.042 (*asterisk*) indicates a significant difference in direct comparison of WT with *Snca* ^−/−^ mice. **e** Quantification of replication competent viral titres (PFU) in the brain at 10 dpi. wt (*n* = 7) and *Snca* ^−/−^ (*n* = 8) male and female littermates were used. **f** Schema of experimental paradigm for bacterial *Salmonella typhimurium* infection, in which WT (*n* = 7) and *Snca*-null (*n* = 8) 8-week-old males and female littermates were inoculated intravenously with 200 colony-forming units (CFU). **g** Mice were sacrificed 5 dpi and spleens were harvested to assay the bacterial load in resected organs (CFU). A number of CFU per spleen of individual mice are plotted by genotype; significance was determined between genotypes using a Mann–Whitney test where *p* = 0.04 (*asterisk*)

We used the reovirus-T3D nasal inoculation paradigm (above; Fig. 4a) to begin to test this. Given that the model requires the infection of newborn pups, we first confirmed the CNS-wide expression of endogenous α-synuclein from post-natal day P1 onward to P42 by a validated, sandwich ELISA (Fig. 4b). Indeed, the protein was detectable at P1, and its concentration rose during the early post-natal brain development and peaked by P21. To determine if endogenous α-synuclein expression altered the outcome of a reovirus-T3D infection, including survival of the host and viral load in the brain, we infected littermates of three distinct genotypes, i.e., wt, *Snca*-heterozygous and *Snca*-null, using our established paradigm (Gauvin et al. 2013) (Fig. 4c). Approximately 24–36 h following birth, mice were inoculated via the nose pad with a dose of 1.7 × 10^5^ plaque-forming units (PFU) of reovirus-T3D. Of note, the experiment was carried out in a blinded manner with respect to the genotype. Remarkably, we found an *Snca* allele dose-dependent effect on the course of illness in these mice, where decreased α-synuclein expression correlated with decreased survival rates (our primary endpoint). *Snca*-null mice (*Snca* ^−/−^) were significantly more affected when compared to wt littermate controls (Fig. 4d), suggesting that α-synuclein may play a role in innate immune function and associated host defense. The survival curve for heterozygous animals fell in between *Snca*-null and wt littermates, but did not show significance. We probed for, but did not observe any sex effect in the outcome of survival.

Unexpectedly, despite the increased mortality observed in *Snca*-null mice, viral titres in the brains at 10 dpi (our second endpoint) did not show any difference in the actual number of infectious virions between wt and *Snca*-null mice (Fig. 4e). We concluded from these results that inoculation efficiency, peripheral epithelial infection, the subsequent systemic dissemination of the virus, and initial brain infection rates were not altered by endogenous *Snca* expression in our paradigm; however, once encephalitis had started, its course was measurably worse in the absence of murine α-synuclein. This suggested that an altered host response to the infection resulted in increased disease severity. Because the expression of α-synuclein is not restricted to neurons (Scherzer et al. 2008; Gray et al. 2014), its protective effects in defending the host against the risk of a RNA virus infection, which leads to lethal encephalitis, may be mediated by more than one cell type including those of the immune system.

### Validation of a role for α-synuclein in innate host defense

To validate our initial findings from the neurotropic reovirus model, we next tested the susceptibility of adult *Snca*-null mice (*vs.* wt animals) to a systemic bacterial infection caused by *S. typhimurium*, which induces lethal sepsis. In this paradigm, mice are injected intravenously with 200 colony-forming units (CFU) of bacteria into the tail vein; animals were euthanized at 5 dpi and bacterial load in the spleen was quantified using a CFU assay (Fig. 4f). In parallel to our viral infection paradigm, mice that lacked *Snca* were less able to control bacterial growth in vivo, and thus showed a significantly increased bacterial load in their spleens compared to their wt littermate controls. Together, these results provided complementary evidence for an innate role for α-synuclein in the host’s response to virulent infections, both systemically and in the brain. Intriguingly, recent studies by Beatman et al. described a similar, protective anti-viral role for murine α-synuclein in vivo (Beatman et al. 2015). Collectively, these findings invite further study to better understand the functions of distinct synuclein proteins (including of its homologues and distinct isoforms) in innate immunity of mammalian hosts. Together with the emerging role of LRRK2 (and Aβ) in innate immunity, they also invite further consideration of a fundamental role for immune mechanisms, as well as of virulent, microbial triggers, in the initiation and/or development of PD (and of other neurodegenerative disorders).

## Discussion

The olfactory system may be one of two pivotal sites, in addition to the gut, for the initiation of PD pathology, as first hypothesized by Braak and Del Tredici over a decade ago (Braak et al. 2003; Del Tredici and Braak 2016). Studying the functional implications of distinct human alleles, including those at the *SNCA* and *MAPT* loci, within the olfactory system (and the enteric nervous system) promises to provide insights into their still elusive, pathological contributions to PD pathogenesis, in particular during the initiation of the disease. The olfactory epithelium (OE) represents an intranasal gateway to the remainder of the olfactory system and the brain, sitting outside the cribriform plate at the interface of the host and his/her environment. The study of the OE has been limited in rodent models of parkinsonism, in large part due to the separation of the nasal epithelia from the brain during routine skull dissections used for histological studies. This key limitation is overcome by applying our method for whole head mounts. The advantage of the holocranohistochemistry protocol described herein, versus the traditional mounting of isolated brains, is that it enables the comprehensive exploration of the whole brain and associated tissues including the OE, cranial nerves (including CN-VII to -XII; not shown), arterial-, venous-, and (g)lymphatic systems, all glands and aspects of the cervical cord (for example), which remain intact when using our skull preparation technique. Therefore, with respect to modeling diseases in mice to pursue genetic leads, this optimized method enables the visualization of the entire olfactory system, which may be important given its relevance to both PD and AD (see Fig. 2 for example). As well, it visualizes the intact respiratory epithelium with its established relevance to chronic, viral infections in mammals and its possible relation to AD susceptibility [(Fig. 3f, h); reviewed by Itzhaki et al. (2016)].

In addition to visualization of protein expression throughout the OE, the advantage of maintaining the olfactory system intact for later histological analysis is its relevance to both physiological (e.g., olfaction) as well as pathogenic (i.e., infection-related) functional readouts in mice. Our goal was to create a tool that allowed us to monitor the olfactory system in a ‘complex disease’ paradigm during gene–environment interaction studies in a rodent, as they possibly relate to the development of PD and AD. In our case, holocranohistochemistry enabled the step-by-step tracking of an environmental pathogen once present within the nasal cavity. Specifically, we observed the entry of a neurotropic reovirus following inoculation of the nose pad via epithelial structures into CN-I (and -V) en route to the brain. In parallel, we could monitor the ensuing immune response by macrophages and microglia (Fig. 3d), aimed at the clearance of infected ORNs. This technique is ammenable to modeling and tracking other environmental exposure events in the nasal cavity of rodents, such as of additional, virulent microbes, or of neurotropic toxins [i.e., metals, including manganese (Racette et al. 2016)]. These could enter the CNS either through the lamina propria underneath the nasal epithelia, or via CN-I and CN-V, or through any of the many structures within the oropharyngeal cavity. Our protocol could also be conducive to tracking preformed protofibril preparations of human α-synuclein and their possible spread from the OE into the brain, as is currently being explored to study murine, prion-type propagation models of PD pathogenesis (see review by Rey et al. 2016).

Similarly, holocranohistochemistry could be used to visualize antigens (and host responses) in pre-clinical studies of active vaccination protocols using Aβ and α-synuclein via intranasal delivery in rodents (e.g., Weiner et al. 2000; Lee and Lee 2016). It also lends itself to exploring the cellular source of neurodegeneration linked proteins, including minute amounts of prion and prion-like proteins, that are being collected in nasal secretions and saliva for current biomarker development purposes (Mollenhauer et al. 2016b; Orru et al. 2014; Beach et al. 2016; Carletti et al. 2017). We have also used the whole head mounting technique to carry out BrdU-labelling studies during regeneration of the injured OE following a reovirus infection, to record post-natal neurogenesis rates in young mouse brains, and to monitor hematopoiesis in the adjacent bone marrow of skull bones. The protocol is also compatible with histological staining for the detection of amyloid-forming proteins including keratin structures by Thioflavin-S and -T (not shown).

Important considerations when applying the holocranohistochemistry protocol include the possibility of epitope alteration (the removal or generation thereof) by formic acid exposure, necessitating judicious confirmation of the specificity of antibody immunoreactivity. This was achieved herein by including sections from gene deleted mice and transgenic animals (i.e., Figs. 2, 3). Although decalcified, cutting adult and pup skulls through serial 5 μm thin sections can also create obstacles, which include decreased adherence to slides, artefacts of tissue tearing, excessive shrinking of select structures (which introduces irregularity of tissue surfaces including of the cortical ribbon), the related exaggeration of tissue space sizes (such as of the subdural space), folding of thin, linear structures during subsequent mounting (such as elements of the cribriform plate and the base of the skull), and the fracturing of teeth in adult mice. For these reasons, increased care is required for the processing of mounted sections during additional ‘antigen retrieval steps’, such as the heating during microwave treatment. This was especially true when pre-treating with proteinase-K prior to immunostaining. As ‘CLARITY’-based imaging studies have expanded the field of neuroscience through its 3D imaging applications (Chung and Deisseroth 2013), thereby markedly enhancing the perception of connectivity within the brain itself, holocranohistochemistry promises to enhance the field of neurodegeneration by providing the anatomical integrity of both intra- and extra-cranial structures that are likely involved in disease development.

Using this technique, we have found that *Snca* and *Mapt* genes are both highly expressed throughout the murine OE, including in dendritic structures of its neurons as well as in axonal bundles of CN-I. Although previously described in humans and explored in select disease processes, metabolic studies of these proteins (as well as of APP) in the healthy OE and CN-I axons of wt animals and genetically modified mice have not been previously carried out. Within ORNs (as throughout the olfactory system), α-synuclein is likely involved in the regulation of specific neuronal functions, i.e., in odour signal transmission. Indeed, we found *SNCA* gene-dose-dependent effects on olfaction in aged PAC-tg (*SNCA* ^A53T^) mice, which correlated with total and soluble oligomeric α-synuclein concentrations measured in parallel by ELISA in the olfactory bulb (and a trend for more insoluble, proteinase-K-resistant species seen in sections of glomeruli and CN-I; Suppl. Fig. 1). However, at what level within the neuroaxis α-synuclein over-expression conferred this effect remains unanswered. Unexpectedly, we found that in addition to the known, pre-synaptic role in neurotransmission, mammalian α-synuclein also played a systemic function in the heretofore overlooked regulation of the susceptibility to pathogens (see below).

We view idiopathic PD in the context of a complex disease, in which genetic susceptibility conspires with an environmental trigger to initiate pathogenesis (Kitada et al. 2012; Schlossmacher et al. 2017). Importantly, we are interested in environmental hits that are microbial in nature, supported in part by the emerging role of PD-linked genes, including *LRRK2*, in the immune system (Hakimi et al. 2011; Gardet et al. 2010; Dzamko et al. 2016). In modeling complex disease processes in mice we have, therefore, restaged a natural course of systemic infection using a nasal delivery paradigm, which initially leads to transient rhinitis and gastrointestinal disease (Gauvin et al. 2013), and monitored neural health in the process.

The abundance of α-synuclein within dendritic structures of ORNs and the presence of proteinase-K-resistant (insoluble) species within axons of CN-I, coupled with its key role in typical PD pathogenesis, led us to test specifically the role of α-synuclein in the susceptibility of a host to virulent infections. For this, we used two infectious paradigms: nasal inoculation with a neurotropic virus in newborn suckling mice (reovirus-T3D; Fig. 4c) and bacterial sepsis following i.v. inoculation of adult mice (*S. typhimurium*; Fig. 4f). To our surprise, in both cases, endogenous, wt α-synuclein was significantly protective. Our viral studies are thus consistent with those published by Beatman et al, who found that *Snca*-null mice were more susceptible to systemic infection by two types of neurotropic RNA viruses (i.e., West Nile virus; Venezuelan equine encephalitis virus, TC83), leading to increased mortality, and in their experimental paradigms, to increased brain viral load (Beatman et al. 2015). Taken together, our respective studies thus found α-synuclein to be protective in four, complementary, well-established in vivo infection paradigms (including in viral and bacterial models) and using three different routes of inoculation for these pathogens (i.e., intravenous, the nose pad, and subcutaneous); these routes are likely to elicit distinct responses by a mammalian host, both within immune cells and other nucleated cells. Collectively, these results unequivocally establish a heretofore unrecognized role for endogenous α-synuclein in anti-microbial defenses in vivo. Whether this function is shared with β-synuclein (an exclusively neuronal protein) and γ-synuclein (expressed also in non-neural cells) remains to be determined.

The mechanism(s) by which α-synuclein is protective in the murine host’s anti-microbial defense remain(s) to be elucidated. Given the short time frame of our two inoculation paradigms before lethality occurs, we favour an important role for α-synuclein in innate host responses, including within the immune system. In our paradigms, it may act indirectly to modulate the function of microglia, neutrophils, macrophages (and less-so of B-cells and T-cells in the adaptive immune system), and/or their development, as previously examined for α-synuclein in ex vivo studies [i.e., (Gardai et al. 2013; Shameli et al. 2016) and as reviewed by Allen Reish and Standaert (2015)]. Alterations in spleen and lymph node structures in *Snca*-null mice have also been reported (Xiao et al. 2014). Alternatively, α-synuclein may function directly within infected cells (i.e., neurons and macrophages) to alter and restrict pathogen uptake, transport, and/or presentation of antigens, lysosomal processing, and thus alter virulence (Beatman et al. 2015; Gardai et al. 2013). Finally, α-synuclein, akin to Aβ protein (Soscia et al. 2010; Kumar et al. 2016), could also act as an anti-microbial peptide (AMP) in direct response to actual exposure to virulent pathogens, as has recently been demonstrated in vitro (Park et al. 2016). Of note, in contrast to the report by Park et al., in our own studies, we did not observe a direct, anti-microbial, and AMP-type effect for either monomeric (recombinant), human α-synuclein, or for dopamine treatment-induced, oligomeric α-synuclein using bacterial cultures of three different organisms (unpublished results). These different outcomes for in vitro studies reported by Park et al., and our own work may be related to technical differences in the experimental design, in bacterial strains employed, and/or in α-synuclein preparations used.

Importantly, the role of α-synuclein in anti-microbial host defenses does not appear to be restricted to the brain, as suggested by results from our bacterial sepsis model (Fig. 4d). Its mechanisms of action are likely pathogen-, cell-type-, and/or immune organ-dependent, and likely include the spleen and, in humans, the appendix (Gray et al. 2014). The spleen is where senescent erythrocytes are continuously degraded, in part by the PD-linked and *GBA*-encoded enzymatic function of acid-β-glucocerebrosidase, and release large amounts of α-synuclein (e.g., (Sardi et al. 2012)). The appendix has been implicated as a ‘storage site’ for healthy, commensal gut organisms, responsible for replenishing the intestinal microbiome subsequent to its depletion in certain disease states (such as severe diarrhea). In this context, it is tempting to speculate that the abundance of α-synuclein present in the appendiceal lamina propria, which we recently described (Gray et al. 2014), has a role in shaping the composition of the intestinal microbiome (Sampson et al. 2016; Scheperjans et al. 2015). To elucidate the mechanisms by which α-synuclein confers a protective role in innate immunity, molecular immunology studies using additional infectious paradigms will be required as well as the employment of nasal, systemic, and intra-cranial delivery methods in adult mice; suitable models that we will employ in such experiments will include *Snca*-null, wt, and PAC-transgene carrying animals that express PD-linked mutations.

Whether the anti-microbial role of α-synuclein in vivo is pathogenetically related to idiopathic PD and other, sporadic synucleinopathies (Ingelsson 2016), remains to be determined; similarly, whether an elevation of (or reduction in) systemic risk for microbial illnesses could underlie the development of PD variants in long-lived humans is currently unclear. Our results, which were inspired by our findings using a new tissue-processing technique, may seem paradoxical in this context, given that the conventional wisdom in the field stipulates that elevated expression and dysregulation of α-synuclein are associated with increased risk of typical, idiopathic PD (Trinh and Farrer 2013). While elevated α-synuclein and its dysregulated metabolism are clearly associated with the propagation and/or progression of the disease (foremost of its microscopically detectable proteinopathy, including in familial cases), perhaps, this is independent of its association with the risk of developing PD, i.e., with the actual initiation of disease. Such a concept would be consistent with the published, but still unexplained, twin findings that there is a significant reduction of total α-synuclein at the protein level in CSF (e.g., (Mollenhauer et al. 2013; Kang et al. 2013) and at the mRNA level in venous blood (Locascio et al. 2015), even in de novo PD patients. Currently, most investigators for the etiology of late-onset PD interpret these biomarker findings (i.e., of low α-synuclein levels) to reflect secondary changes in response to a disease process that is already underway. Alternatively, we propose that a possible positive association between systemic reduction in α-synuclein (i.e., as genomic risk) and increased susceptibility by a host to develop PD should be considered as well (Schlossmacher et al. 2017).

Furthermore, several reports have recently demonstrated a mild, but consistent reduction for tau proteins in CSF from subjects with typical PD [reviewed in Mollenhauer et al. (2016a)]. Although we did not explore the role of tau in innate immune defenses here, future studies should address whether variants at the *MAPT* locus, which alter the risk to PD (Elbaz et al. 2011; Oikawa et al. 2016; Rousseaux et al. 2016; Edwards et al. 2010), also change the susceptibility in mammals regarding infections by virulent xenobiotics, or alternatively, modify their course (as does a mutant *Snca* genotype). Of possible relevance is the fact that microtubule-associated proteins (MAPs) are effectively exploited by viruses when trafficking within infected cells (reviewed in Portilho et al. 2016).

Theoretically, variants at the *SNCA* and *MAPT* loci, as identified by GWAS, could alter the expression levels of their encoded proteins, either systemically or at select sites within the nervous system, thereby modifying host responses to environmental pathogens [as part of each subject’s ‘exposome’ risk (Rappaport 2016)]. In doing so, α-synuclein and tau could contribute to the overall susceptibility of a human subject to develop PD. If that were to be the case, then distinct variants at the *SNCA* and *MAPT* loci would be involved in a whole spectrum of disease-promoting mechanisms, i.e., from co-regulating overall susceptibility to PD at a site where gene–environment interactions occur, to participating in the ensuing tissue responses that propagate disease, and in doing so, to ultimately co-regulating the phenotypic expressivity of an α-synuclein-related disorder.

As recently vigorously debated, disease progression in PD could be explained by a prion-like propagation of α-synuclein itself across synapses (reviewed in: Walsh and Selkoe 2016; Rey et al. 2016); alternatively, an ‘initiating factor’, i.e., an environment-derived pathogen that leads to α-synuclein dysregulation within affected neurons (rather than misfolded α-synuclein species themselves) could gradually propagate across synapses. This could also explain the “spread of Lewy pathology” from the host to grafted cells in recipients of fetal tissue transplants (e.g., Chu and Kordower 2010). Of note, changes in α-synuclein metabolism, including the up-regulation of the gene and its post-translational modification (phosphorylation; oligomerization), have been shown to occur downstream of microbial insults in vivo (Beatman et al. 2015; Jang et al. 2009; Chen et al. 2016). Both aforementioned theories remain speculative (as could be a combination of both scenarios). Because the presence of fibrils has not yet been established in extracellular fluid spaces of human brain, each of these different models for disease development warrants further consideration.

Of note, we saw no evidence of α-synuclein accumulation or enhanced oligomer (or fibril) formation at 24 day post-reovirus-T3D inoculation in skull sections of those mice that had survived their encephalitis; however, as discussed above, the holocranohistochemistry technique may not be sensitive enough to detect mild-to-moderate changes of this long-lived protein (Fishbein et al. 2014) by microscopy alone, and the time course for any dysregulation to occur may have been too short (i.e., between the start of encephalitis at 9 dpi and euthanasia at 24 dpi). As such, and to further explore the relevance of our findings to PD etiology and/or pathogenesis, in future studies, we will monitor changes in α-synuclein metabolism, neuropathology, and disease-relevant behavioural outcomes in response to chronic, non-lethal infection paradigms in adult mice. This includes using tg mice that express disease-linked α-synuclein variants (e.g., p.A30P and p.E46K mutants) and, as shown here, elevated copy numbers of *SNCA* alleles that encode the wt human protein under its physiological promoter (e.g., Kuo et al. 2010; Fig. 2 and Suppl. Fig. 1).

In support of a complex disease model for typical PD, we find it tantalizing that variants of another PD gene, *LRRK2,* have recently been found to alter the course of microbial infections in select paradigms from experimental investigations; these were conducted in immune cells, mice, and humans to better understand its association with three complex diseases (i.e., PD, Crohn’s and leprosy) (Hakimi et al. 2011; Ness et al. 2013; Liu et al. 2011; Fava et al. 2016; Dzamko et al. 2016).

Regardless of the possible association of a role for α-synuclein in the host’s anti-microbial defense with any relevance to actual PD initiation, our findings raise important implications for a range of clinical research activities that focus on the lowering of total (or oligomeric species) of α-synuclein as a therapeutic strategy (Masliah et al. 2005; Lee and Lee 2016; Schenk et al. 2017). Because, ultimately, such an intervention would require long-term administration of a drug in elderly patients (and, therefore, in less immune-privileged subjects), it may impose unintended, adverse effects, namely by elevating their rate of contracting virulent infections, or alternatively, by experiencing worse outcomes thereof. Hence, ongoing monitoring of systemic health will be important to add to that of nervous system function.

In summary, we believe that our optimized protocol for carrying out the method of holocranohistochemistry promises to inform future studies in the following three ways: one, to better model the complex interactions between environmental pathogens and distinct risk alleles (e.g., *SNCA*, *MAPT,* and *APP*) in the nasal cavity and oropharynx. Hopefully, this will provide more insights into the actual initiation of late-onset neurodegenerative disorders in humans; two, to delineate how natural infections, such as those in the nasal cavity, will alter the metabolism of proteins that are encoded by disease-linked risk alleles; and three, to better design studies in laboratory animals that capture systemic health outcomes as well as nervous system readouts when studying interactions between the host’s genome and his/her exposome.

## Electronic supplementary material

Below is the link to the electronic supplementary material.

Supplementary material 1 (PDF 647 kb) **Supplemental Fig.** **1: Elevated expression of** α**-synuclein in the nervous system in human** ***SNCA*** **allele-transgenic mice leads to olfactory dysfunction.** Dbl-PAC- tg(*SNCA* ^A53T^)^+/+^; *Snca* ^−/−^ FBV/Nx129S6 mice [4 genomic insertions of the PAC; Homo] (Kuo et al. 2010) were crossed with *Snca* ^−/−^ mice on the same genetic background (Kuo et al. 2010) to create PAC-tg(*SNCA* ^A53T^)^+/−^ FBV/Nx129S6 mice [2 genomic insertions of the PAC; Het]. *SNCA* gene-dosage-dependence was determined by comparison of age-matched ‘Homo’ *versus* ‘Het’ mice. **(A)** Representative image of H&E stained mouse olfactory system from a whole skull mount; highlighted are the ethmoid sinus (ES), glomeruli (GL), olfactory bulb (OB), and CN-I (asterix). **(B-I)** Immunostaining-based microscopy with monoclonal antibody (OT21C) reveals ‘total- (-ProtK)’ and ‘proteinase-K-resistant (+ProtK)’ human α-synuclein species using sagittal sections of whole skull mounts from 8 month-old ‘Het’; (**B-C**) or ‘Homo’ (**E–F**) mice. Note the trend for reduced signal in staining of human α-synuclein in the neuropil of the OB and CN-I (asterix) in ‘Het’ animals with two insertions of the PAC-*SNCA* allele. In contrast, ‘Homo’ mice with four insertions show relative proteinase-K-resistance of human α-synuclein in most of the GL and CN-I. **(D)** *Snca* ^−/−^ mice and **(H-I)** human brain tissue from a patient with dementia with Lewy body collected at autopsy were used as negative and positive Ab controls, respectively. **(G)** Similar staining was observed when the human α-synuclein antibody hSA4 was used in parallel on proteinase-K treated ‘Homo’ mouse sections. All images are representative of a minimum of n = 3/genotype/treatment. **(J)** ELISA-based quantification of total and **(K)** soluble oligomeric α-synuclein (aSyn) in the olfactory bulb (OB) and forebrain homogenates of 14–15-month-old ‘Homo’ (n = 5) and ‘Het’ (n = 8) female mice using the ELISA platforms described (Vaikath et al. 2015). **(L,M)** Assessment of *SNCA* gene-dose-dependent effects on olfactory function in 16-month-old ‘Homo’ and ‘Het’ mice). **(L)** We did not detect *SNCA* gene-dose-dependent differences in the Odour Detection test. Data are represented as the mean percent (%) time sniffing odour (olfactory cue; anise; and vanilla extracts) *versus* water where 50% time represents ‘chance’. **(M)** Increased α-synuclein load correlated with a significant deficit in the odour habituation/dishabituation test, specifically in the social cue **(M)**; n = 12 per genotype (M = F), *p ≤ 0.05 by repeated-measures ANOVA
