# Supplementary figures and images for: Holocranohistochemistry enables the visualization of α-synuclein expression in the murine olfactory system and discovery of its systemic anti-microbial effects
Source: J Neural Transm (Vienna). 2017 May 5;124(6):721–38. doi: 10.1007/s00702-017-1726-7 (PMC5446848; doi:10.1007/s00702-017-1726-7)

## Supplemental Figure 1

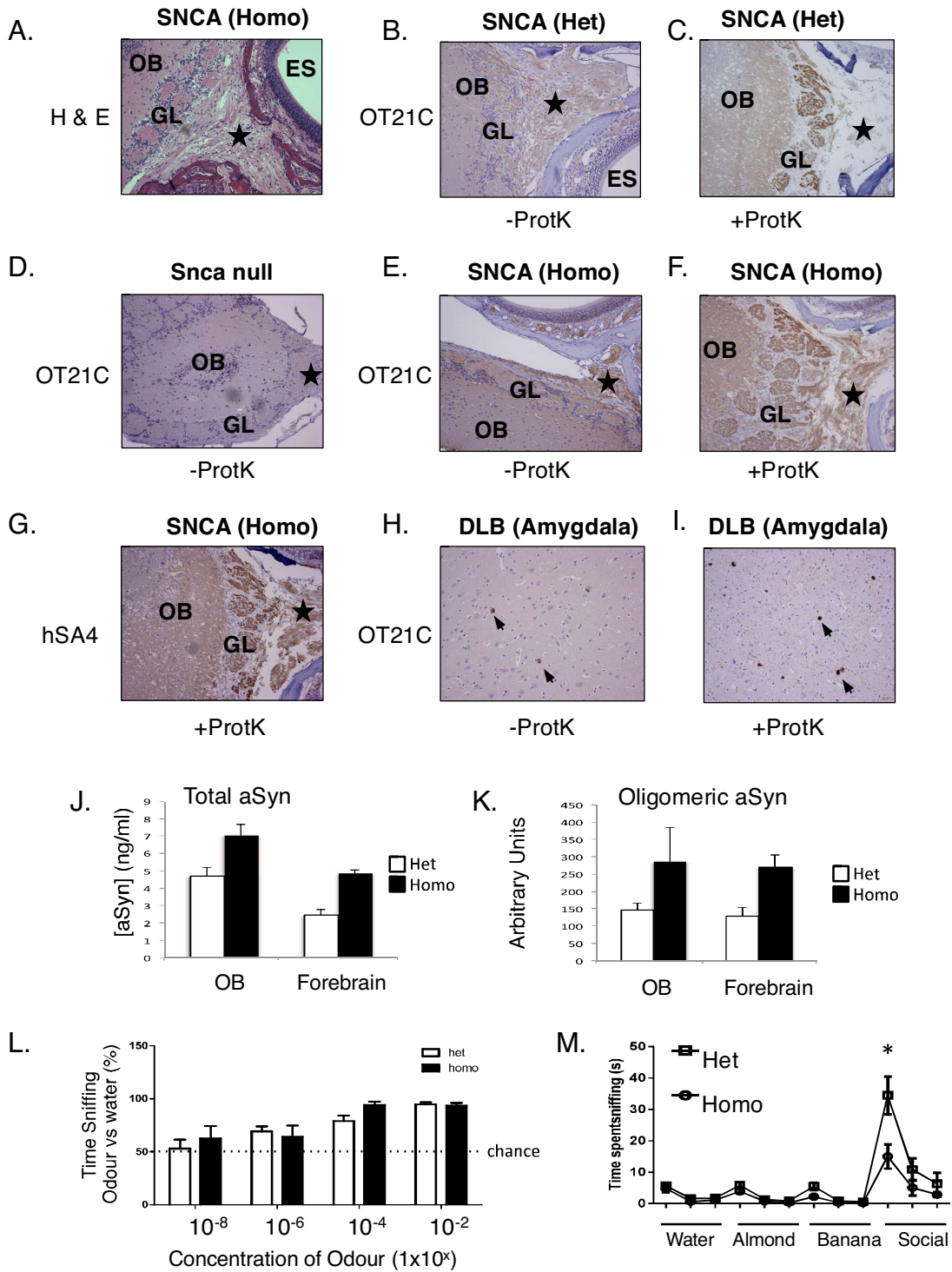

Supplement: Supplementary file 1 — Supplementary material 1 (PDF 647 kb) Supplemental Fig. 1: Elevated expression of α-synuclein in the nervous system in human SNCA allele-transgenic mice leads to olfactory dysfunction. Dbl-PAC- tg(SNCA A53T)+/+; Snca −/− FBV/Nx129S6 mice [4 genomic insertions of the PAC; Homo] (Kuo et al. 2010) were crossed with Snca −/− mice on the same genetic background (Kuo et al. 2010) to create PAC-tg(SNCA A53T)+/− FBV/Nx129S6 mice [2 genomic insertions of the PAC; Het]. SNCA gene-dosage-dependence was determined by comparison of age-matched ‘Homo’ versus ‘Het’ mice. (A) Representative image of H&E stained mouse olfactory system from a whole skull mount; highlighted are the ethmoid sinus (ES), glomeruli (GL), olfactory bulb (OB), and CN-I (asterix). (B-I) Immunostaining-based microscopy with monoclonal antibody (OT21C) reveals ‘total- (-ProtK)’ and ‘proteinase-K-resistant (+ProtK)’ human α-synuclein species using sagittal sections of whole skull mounts from 8 month-old ‘Het’; (B-C) or ‘Homo’ (E–F) mice. Note the trend for reduced signal in staining of human α-synuclein in the neuropil of the OB and CN-I (asterix) in ‘Het’ animals with two insertions of the PAC-SNCA allele. In contrast, ‘Homo’ mice with four insertions show relative proteinase-K-resistance of human α-synuclein in most of the GL and CN-I. (D) Snca −/− mice and (H-I) human brain tissue from a patient with dementia with Lewy body collected at autopsy were used as negative and positive Ab controls, respectively. (G) Similar staining was observed when the human α-synuclein antibody hSA4 was used in parallel on proteinase-K treated ‘Homo’ mouse sections. All images are representative of a minimum of n = 3/genotype/treatment. (J) ELISA-based quantification of total and (K) soluble oligomeric α-synuclein (aSyn) in the olfactory bulb (OB) and forebrain homogenates of 14–15-month-old ‘Homo’ (n = 5) and ‘Het’ (n = 8) female mice using the ELISA platforms described (Vaikath et al. 2015). (L,M) Assessment of SNCA gene-dose- [file 702_2017_1726_MOESM1_ESM.pdf]
